# Probiotics Enhance *Coilia nasus* Growth Performance and Nutritional Value by Regulating Glucolipid Metabolism via the Gut–Liver Axis

**DOI:** 10.3390/ijms252212196

**Published:** 2024-11-13

**Authors:** Qi Mang, Jun Gao, Quanjie Li, Yi Sun, Gangchun Xu, Pao Xu

**Affiliations:** 1 Wuxi Fisheries College, Nanjing Agricultural University, Wuxi 214081, China; mangq@cafs.ac.cn; 2 Key Laboratory of Freshwater Fisheries and Germplasm Resources Utilization, Ministry of Agriculture, Freshwater Fisheries Research Center, Chinese Academy of Fishery Sciences, Wuxi 214081, China; gaojun@ffrc.cn (J.G.); liqj@ffrc.cn (Q.L.); sunyi@ffrc.cn (Y.S.)

**Keywords:** probiotics, growth performance, nutritional value, glucolipid metabolism, gut–liver axis, *Coilia nasus*

## Abstract

Large-scale intensive feeding triggered reduced growth performance and nutritional value. Exogenous probiotics can promote the growth performance and nutritional value of fish through improving the intestinal microbiota. However, detailed research on the correlation between the intestinal microbiota, growth performance, and nutritional value remains to be elucidated. Therefore, we performed metagenomic and metabolomic analysis to investigate the effects of probiotic addition to basal diet (1.0 × 10^8^ CFU/g) (PF) and water (1.0 × 10^8^ CFU/g) (PW) on the growth performance, muscle nutritional value, intestinal microbiota and their metabolites, and glucolipid metabolism in *Coilia nasus*. The results showed that FBW, BL, and SGR were enhanced in PF and PW groups. The concentrations of EAAs, TAAs, SFAs, MUFAs, and PUFAs were increased in PF and PW groups. Metagenomic and metabolic analyses revealed that bacterial community structure and metabolism were changed in the PF and PW groups. Moreover, adding probiotics to diet and water increased SCFAs and bile acids in the intestine. The gene expression associated with lipolysis and oxidation (*hsl*, *pparα*, *cpt1*, and *acadm*) and glycolysis (*gck* and *pfk*) was upregulated, while the gene expression associated with lipid synthesis (*srebp1*, *acc*, *dgat*, and *elovl6*) and gluconeogenesis (*g6pca1*, *g6pca2*, and *pck*) was downregulated in the liver. Correlation analysis displayed that hepatic glucolipid metabolism was regulated through the microbiota–gut–liver axis. Mantel test analysis showed that growth performance and muscle nutritional value were improved by the gut–liver axis. Our findings offered novel insights into the mechanisms that underlie the enhancement of growth performance and nutritional value in *C. nasus* and other fish by adding probiotics.

## 1. Introduction

With the continuous growth of the global population and increasing demand for healthy diets, the demand for high-quality protein is increasing day by day [1]. Fish are important economic and nutritional resources, providing high-quality protein for humans [2]. However, intensive aquaculture is facing many challenges, such as low feed efficiency and environmental issues, which threaten food safety and the quality of fish [2]. It is essential for aquaculture to enhance fish growth performance and improve meat quality [3]. In recent years, probiotics have shown great potential in enhancing growth performance and improving meat quality [2].

Probiotics, considered beneficial microorganisms that promote gut health, play an important regulatory role in humans, livestock [4], poultry [5], and aquatic animals [1]. Probiotics have a positive impact on the growth performance of animals. Previous research has proved that probiotics can increase feed utilization, improve feed conversion efficiency, and thereby promote the growth performance of aquatic animals. Host gut-derived *Bacillus* supplementation significantly improved growth performance in hybrid grouper (♀*Epinephelus fuscoguttatus* × ♂*E. lanceolatus*) [6]. *Lactobacillus acidophilus* and *L. bulgaricus* supplementation significantly enhanced the growth performance in *Oncorhynchus mykiss* [7]. The inclusion of dietary immunobacterin (IMB, 1.5 g/kg) notably enhanced growth performance, feed intake, and secretion of growth hormone in Nile tilapia (*Oreochromis niloticus*) [8]. Besides improving growth performance, probiotics have also displayed positive effects on enhancing muscle quality. It has been reported that probiotics can promote muscle protein synthesis, increase the diameter and quantity of muscle fibers, and enhance muscle fatty acid and amino acid content [9]. They can also regulate the ratio of muscle adipose tissue and reduce fat deposition in muscles [10]. The inclusion of *Clostridium butyricum* in the diet exhibited positive effects on performance, lipid metabolism, and meat quality, as well as the composition of amino acids and fatty acids [11].

Recently, increasing research has reported that probiotics alter the microbial community structure and metabolites in the gut to regulate the gut–liver axis, promote the regulation of glucolipid metabolism, and improve the growth performance and muscle quality of fish [12,13,14]. Recent studies have shown that probiotics interact with intestinal epithelial cells and modulate the composition of gut microbiota, thereby influencing nutrient absorption and metabolism in the intestine [15]. Furthermore, these microorganisms can produce bioactive metabolites that directly or indirectly impact the host’s metabolic processes [16,17]. Metabolites such as short-chain fatty acids (SCFAs) produced by probiotics affect liver glucolipid metabolism through the gut–liver axis signal conduction [18], including enhancing insulin sensitivity, lowering blood glucose levels, and promoting fat oxidation [19,20].

*Coilia nasus* is a delicious and valuable fish. At present, the growing development of intensive feeding has seriously threatened their growth performance and nutritional value. A previous study reported that probiotic supplementation could promote growth performance and appetite regulation and inhibit inflammation factors [21]. Nevertheless, limited studies focused on the effects of probiotic supplementation on the gut–liver axis, and there is limited use of metagenomic and metabolic analysis to establish correlations between fish metabolomes and gut microbiota. The present study aims to explore the regulatory mechanism of probiotics promoting growth performance and nutritional value through the regulation of glucolipid metabolism via the gut–liver axis. Our findings will provide new strategies and methods for aquaculture.

## 2. Results

### 2.1. Effects of Probiotic Supplementation on Growth Performance

The growth performance is significantly different in *C. nasus* among different treatment groups (Table 1). At each time point, the FBW and SGR of the PF group were significantly elevated compared to the C group (*p* < 0.05). The FBW of the PW group was significantly higher than the C group at 30 days (*p* < 0.05). The BL of the PW group was significantly increased compared to the C group at 90 days (*p* < 0.05). The SGR of the PW group was significantly enhanced compared to the C group at 30 and 90 days (*p* < 0.05). However, no significant differences were observed in VSI, HSI, and CF at each time point (*p* < 0.05).

### 2.2. Effects of Probiotic Supplementation on Biochemical Indexes of Serum

**Figure 1:**
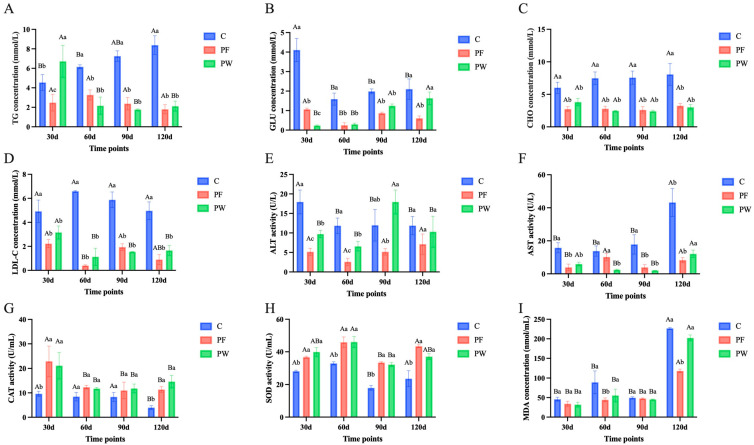
Biochemical indexes of *C. nasus* serum treated by probiotic supplementation. Triglyceride (TG) (**A**), glucose (GLU) (**B**), cholesterol (CHO) (**C**), low-density lipoprotein cholesterol (LDL-C) (**D**), alanine aminotransferase (ALT) (**E**), aspartate aminotransferase (AST) (**F**), catalase (CAT) (**G**), superoxide dismutase (SOD) (**H**), and malondialdehyde (MDA) (**I**). The values were shown in means ± SE. *n* = 9 per treatment group. Different capital letters indicate significant differences among different time points in the same groups at *p* < 0.05. Different lower-case letters indicate significant differences between different groups at the same time point at *p* < 0.05.

After adding probiotics for 30, 60, 90, and 120 days, the concentrations of serum CHO, TG, GLU, LDL-C, MDA, and the activities of ALT, AST, CAT, and SOD in C. nasus were measured (Figure 1). At 60, 90, and 120 days, the TG levels in the PF and PW groups were significantly decreased compared to the C group (*p* < 0.05) (Figure 1A). Compared to the C group, the GLU levels in the PF and PW groups were significantly reduced at 30, 60, and 90 days (*p* < 0.05) (Figure 1B). The CHO and LDL-C levels in the PF and PW groups were lower than the C group at each time point (*p* < 0.05) (Figure 1C,D). The ALT and AST activity in the PF group were significantly lower than the C group at each time point (*p* < 0.05) (Figure 1E,F). The CAT and SOD activity in both the PF and PW groups were significantly increased compared to the C group at each time point (*p* < 0.05) (Figure 1G,H). The MDA levels in the PF group were significantly lower compared to the C group at 60 and 120 days (Figure 1I).

### 2.3. Effects of Probiotic Supplementation on Hydrolyzed Amino Acids (HAAs) and Free Fatty Acids (FAAs) of Muscle

A total of 19 hydrolyzed amino acids were detected, including 11 NEAA and 8 EAA (Table 2). The composition of HAA types in the muscle tissue of *C. nasus* was similar among the three different treatment groups. The TAA levels in the PF group were significantly bettered compared to the C group at 30, 60, and 90 days (*p* < 0.05); however, they were significantly diminished compared to the C group at 120 days (*p* < 0.05). The TAA levels in the PW group were increased compared to the C group at 30, 90, and 120 days (*p* < 0.05). The EAA levels in the PF group were significantly augmented compared to the C group at 30 days (*p* < 0.05); however, they were significantly attenuated compared to the C group at 90 and 120 days (*p* < 0.05). The EAA levels in the PW group were significantly higher than the C group at 30, 90, and 120 days (*p* < 0.05). Furthermore, some important and limited amino acids were enhanced in the PF and PW groups. For example, Lys levels in the PF group were significantly higher than the C group at 90 and 120 days (*p* < 0.05). Thr levels in the PF group were significantly higher than those in the C group at 30, 60, and 90 days (*p* < 0.05). Arg levels in the PF and PW groups were significantly higher than those in the C group at 60 and 90 days (*p* < 0.05).

A total of 35 free fatty acids were detected in the muscle tissue of *C. nasus*, including 16 SFAs, 9 MUFAs, and 10 PUMAs (Table 3). The composition and structure of fatty acids were similar among the different groups. PF and PW groups showed significantly higher levels of SFA than the C group at 60 and 90 days. In the PF group, MUFA and PUFA were significantly increased compared to the C group at 90 days. In the PW group, PUFA levels were significantly bettered compared to the C group at 60 and 120 days (*p* < 0.05). Additionally, both PF and PW groups exhibited significantly higher levels of C20:5 (EPA) than the C group at 60 and 90 days (*p* < 0.05). Moreover, the PW group showed significantly higher levels of C22:6 (DHA) than the C group at 60, 90, and 120 days (*p* < 0.05), while the PF group exhibited significantly higher levels of C22:6 (DHA) than the C group at 90 days (*p* < 0.05).

### 2.4. Metagenomic and Metabolomic Analysis of Intestinal Contents

Statistical analysis of clean reads and assembly results of the C, PF, and PW groups is shown in Tables S3 and S4. PCoA (Figure S1) and α diversity (Figure S2) analyses showed no significant differences. Gut microbiota community structure analysis showed increased relative abundance of Spirochaetota in the PF group at 30 days, while the relative abundance of Proteobacteria and Bacteroidota was risen in the PW group at 30 days (Figure S3A). Additionally, the relative abundance of Firmicutes was augmented in the PF group at 60 days (Figure S3B). The relative abundance of Firmicutes was increased in the PW group at 90 days (Figure S3C). In the PF and PW groups, the relative abundance of Firmicutes increased at 120 days, while the relative abundance of Spirochaetota was decreased at 120 days (Figure S3D). CAZy annotation analysis displayed that the relative abundance of CAZyme in the PF group was higher than the other two groups at each time point (Figure S4). KEGG enrichment analysis indicated that the relative abundance of metabolism-related genes in the PF group was higher than the other two groups at each time point (Figure S4).

**Figure 2:**
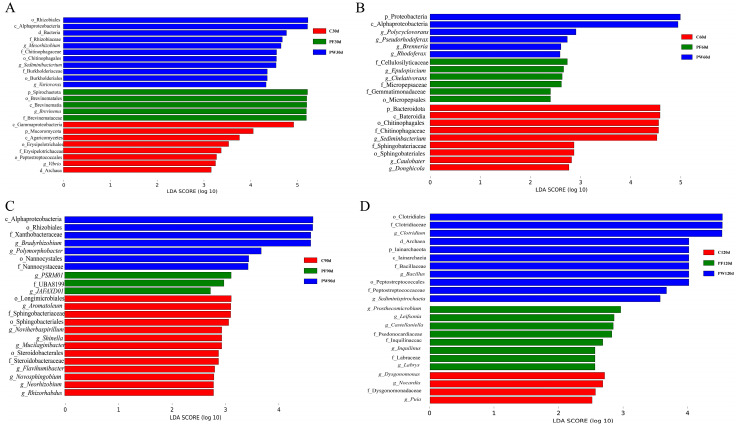
LEfSe analysis of *C. nasus* intestinal microbiota affected by probiotic supplementation at 30d (**A**), 60d (**B**), 90d (**C**), and 120d (**D**). The length of the histogram represents the influence of different species (LDA score).

LEfSe was used to analyze microbial differences. The histogram of LDA value distribution indicates that there are microbial taxa with significant differences in the gut microbiota of *C. nasus* among different groups and time points. As shown in Figure 2A, at 30 days, o_Rhizobiales, c_Alphaproteobacteria, and f_Rhizobiaceae showed significant enrichment in the PW group, while p_Spirochaetota, o_Brevinematales, and c_Brevinematia displayed significant enrichment in the PF group, and c_Gammaproteobacteria, p_Mucoromycota, and c_Agaricomycetes presented significant enrichment in the C group. As shown in Figure 2B, at 60 days, p_Proteobacteria, c_Alphaproteobacteria, and g_Polycyclovorans revealed significant enrichment in the PW group, while f_Cellulosilyticaceae, g_Epulopiscium, and *g_Chelativorans* exhibited significant enrichment in the PF group, and significant enrichment of p_Bacteroidota, c_Bateroidia, and o_Chitinophagales was observed in the C group. As shown in Figure 2C, at 90 days, significant enrichment of c_Alphaproteobacteria, o_Rhizobiales, and f_Xanthobacteraceae was found in the PW group, while significant enrichment of g_PSRM01, f_UBA8199, and g_JAFAXD01 was discovered in the PF group, and significant enrichment of o_Longimicrobiales, g_Aromatoleum, and f_Sphingobacteriaceae was shown in the C group. As shown in Figure 2D, at 120 days, significant enrichment of o_Clotridiales, f_Clotridiaceae, and *g_Clotridium* was displayed in the PW group, while significant enrichment of *g_Prosthecomicrobium*, *g_Leifsoniag*, and *g_Castellaniella* was presented in the PF group, and f_PHOS_HE28, g_PHOS_HE28, and f_UXAT02 were significantly enriched in the C group.

**Figure 3:**
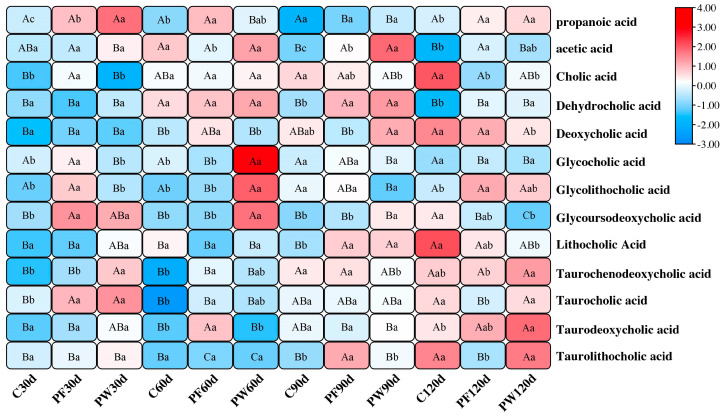
Heatmap of concentration of differential metabolites in *C. nasus* intestinal contents treated by probiotic supplementation. Different capital letters indicate significant differences among different time points in the same groups at *p* < 0.05. Different lower-case letters indicate significant differences between different groups at the same time point at *p* < 0.05.

The OPLS-DA analysis showed not significant results (Figure S5). However, based on *p* value < 0.05, Foldchange > 1, and VIP > 1, the differential metabolites were identified, and the numbers of them among the groups were shown in Table S5. Based on their potential function, SCFAs and bile acids, including their derivatives, can regulate glucolipid metabolism. We selected 2 types of short-chain fatty acids and 11 types of bile acids and their derivatives for analysis. As shown in Figure 3, Compared to the C group, propanoic acid in the PF and PW groups was significantly elevated at 30 days (*p* < 0.05). Acetic acid in the PF and PW groups was also significantly increased compared to the C group at 30 days (*p* < 0.05). Cholic acid and deoxycholic acid in the PF group were significantly higher compared to the C group at 30 days and 60 days, respectively (*p* < 0.05). Dehydrocholic acid in the PF and PW groups was significantly enhanced compared to the C group at 90 days and 120 days (*p* < 0.05). Glycocholic acid, glycolithocholic acid, and glycoursodeoxycholic acid in the PF and PW groups were significantly promoted compared to the C group at 30 days and 60 days, respectively (*p* < 0.05). Lithocholic acid in the C group was significantly decreased compared to the PF and PW groups at 90 days (*p* < 0.05). Taurochenodeoxycholic acid in the C group was significantly higher than the PF and PW groups at 60 days and 30 days, respectively (*p* < 0.05). Taurocholic acid in the C group was significantly reduced compared to the PF and PW groups at 30 days (*p* < 0.05). Taurodeoxycholic acid in the C group was significantly inhibited compared to the PF and PW groups at 60 days and 120 days, respectively (*p* < 0.05). Taurolithocholic acid in the C group was depressed compared to the PF group at 90 days (*p* < 0.05).

### 2.5. Analysis of Genes Related to Glucolipid Metabolism in Liver

**Figure 4:**
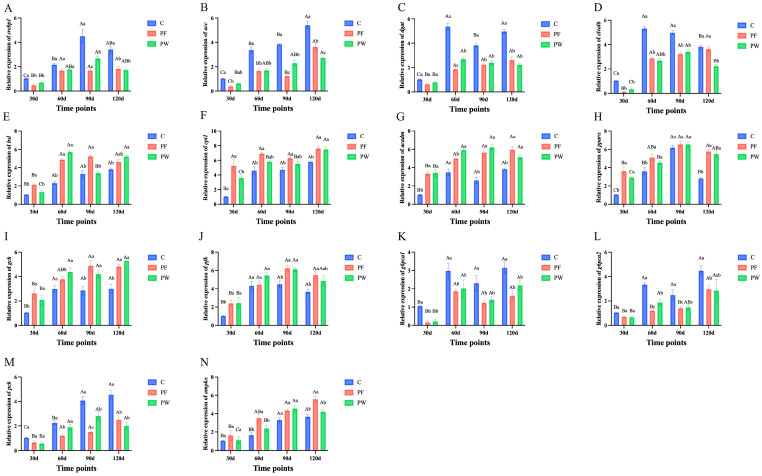
Relative expression of lipid and glucose metabolism in *C. nasus* liver treated by probiotic supplementation. Genes related to lipid synthesis: *srebp1* (**A**), *acc* (**B**), *dgat* (**C**), and *elvol6* (**D**); genes related to lipidolysis and lipid oxidation: *hsl* (**E**), *cpt1* (**F**), *acadm* (**G**), and *pparα* (**H**); genes related to glycolysis: *gck* (**I**) and *pfk* (**J**); genes related to gluconeogenesis: *g6pca1* (**K**), *g6pca2* (**L**), and *pck* (**M**); and genes related to energy metabolism: *ampkα* (**N**). The results were shown in means ± SE. *n* = 9 per treatment group. Different capital letters indicate significant differences among different time points in the same groups at *p* < 0.05. Different lower-case letters indicate significant differences between different groups at the same time point at *p* < 0.05.

Expression of *srebp1* (Figure 4A), *acc* (Figure 4B), *dgat* (Figure 4C), and *elvol6* (Figure 4D) involved in lipid synthesis showed a decreased trend in the PF and PW groups. Expression of *hsl* (Figure 4E), *pparα* (Figure 4F), *cpt1* (Figure 4G), and *acadm* (Figure 4H) involved in lipolysis and lipid oxidation showed an increased trend in the PF and PW groups. Expression of *gck* (Figure 4I) and *pfk* (Figure 4J) involved in glycolysis showed a decreased trend in the PF and PW groups, while expression of *g6pca1* (Figure 4K), *g6pca2* (Figure 4L), and *pck* (Figure 4M) involved in gluconeogenesis showed an increased trend in the PF and PW groups. Expression of *ampkα* regulating glucolipid metabolism showed an increased trend in the PF and PW groups (Figure 4N).

### 2.6. Integration Analysis by Pearson Correlation and Mantel Test

**Figure 5:**
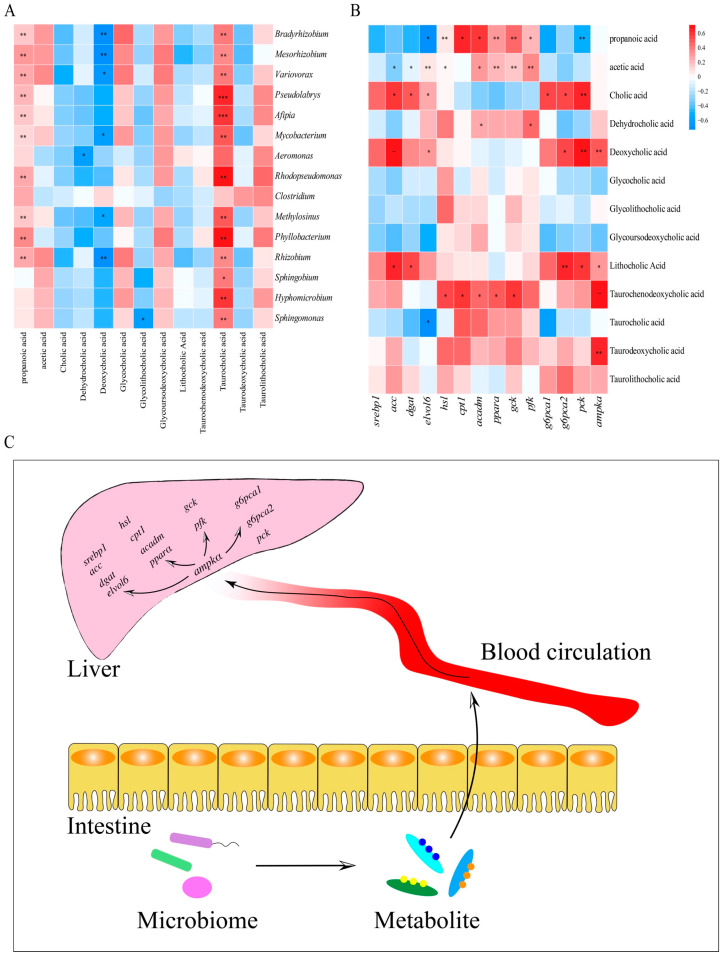
Correlation analysis of intestinal microbiome and microbial metabolites (**A**); correlation analysis of microbial metabolites and genes related to lipid and glucose metabolism (**B**); microbial regulation of gut–liver axis (**C**). The difference was considered statistically significant when * *p* < 0.05, ** *p* < 0.01, or *** *p* < 0.001.

Correlation analysis between the top 15 (relative abundance) microbial taxa (genus level) and selected differential metabolites was performed. As showed in Figure 5A, propanoic acid and taurocholic acid exhibited significant positive correlations with the majority of microbial taxa (genus level); deoxycholic acid is significantly negatively correlated with *g_Bradyrhizobium*, *g_Mesorhizobium*, *g_Variovorax*, *g_Mycobacterium*, and *g_Methylosinus*; and glycolithocholic acid is significantly negatively correlated with *g_Sphingomonas*. Moreover, correlation analysis between the genes related to lipid and glucose metabolism and selected differential metabolites was performed. As displayed in Figure 4B, cholic acid, deoxycholic acid, and lithocholic acid are significantly positively correlated with genes related to lipid synthesis (*acc*, *dgat*, and *elvol6*); propanoic acid and taurochenodeoxycholic acid exhibited significant positive correlation with genes related to lipid oxidative decomposition (*hsl*, *pparα*, *cpt1*, and *acadm*); deoxycholic acid and taurochenodeoxycholic acid presented significant positive correlation with genes related to glycolysis (*gck* and *pfk*); cholic acid, deoxycholic acid, and lithocholic acid are significantly positively correlated with genes related to gluconeogenesis (*g6pca1*, *g6pca2*, and *pck*).

**Figure 6:**
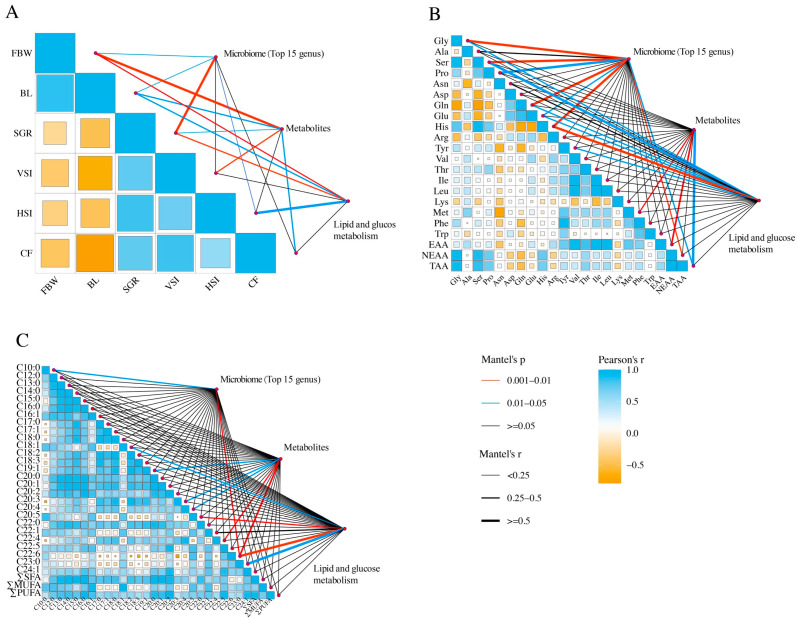
Correlation analysis of the metabolite, microbiome, and glucolipid metabolism and growth performance (**A**), hydrolyzed amino acid (**B**), and free fatty acids (**C**). The color of the square in the matrix denotes the value of the coefficient; blue square indicates positive correlations, and orange square represents negative correlations. The size of the square in the matrix denotes the value of statistical differences. The lines outside the matrix reveal the correlation, with thicker lines representing more significant correlations. The color of the line denotes the value of statistical differences.

The Mantel test was used for correlation analysis of growth performance, HAAs, FFAs, metabolites, microbiome (top 15 genus), and glucolipid metabolism. As shown in Figure 6A, SGR showed the significant correlation (*p* < 0.01) and high correlation coefficient (r > 0.5) to the microbiome. FBW showed the significant correlation (*p* < 0.01) and high correlation coefficient (r > 0.5) to metabolites. SGR and FBW showed the significant correlation (*p* < 0.01) to glucolipid metabolism. As shown in Figure 6B, Gly, Ser, Gln, and His displayed the significant correlation (*p* < 0.05) and high correlation coefficient (r > 0.5) to microbiome. TAA displayed the significant correlation (*p* < 0.05) and high correlation coefficient (r > 0.5) to metabolites. Met and EAA displayed the significant correlation to metabolites (*p* < 0.01). Ser and His displayed the significant correlation (*p* < 0.05) and high correlation coefficient (r > 0.5) to glucolipid metabolism. As shown in Figure 6C, C20:5 (EPA) displayed the significant correlation (*p* < 0.01) to glucolipid metabolism. C22:6 (DHA) displayed the significant correlation (*p* < 0.01) to metabolites, microbiome (top 15 genus), and glucolipid metabolism.

## 3. Discussion

In recent years, probiotics have been widely applied for improving growth performance and nutritional value in aquaculture. *C. carpio* provided with *Enterococcus casseliflavus* in diet enhanced weight gain and specific growth rate, while depressed feed conversion ratio [22]. The similar results also occurred in Caspian white fish (*Rutilus frisii kutum*) [23]. *O. niloticus* fed with *L. rhamnosus* improved weight gain, protein efficiency ratio, SGR, and condition factor [24]. In the present study, probiotic supplementation in diet and water significantly promoted body weight and length, as well as SGR in *C. nasus*. These results indicated that adding probiotics significantly promoted the growth performance of *C. nasus*. Recently, growing numbers of studies are focusing on improving fish meat quality. Muscle nutritional values are becoming crucial indicators of fish meat quality [25]. The content of amino acids and fatty acids directly reflects the nutritional value [26]. In this study, adding probiotics significantly increased the content of EAA and TAA in *C. nasus* muscle. The composition and ratio of amino acids in muscle determine the quality of protein [27]. The umami taste of fish meat is primarily determined by the content of umami amino acids, including glutamate, arginine, glycine, and aspartic acid [28]. In this study, adding probiotics significantly increased the content of glutamate and arginine in *C. nasus* muscle, which indicated that adding probiotics could enhance umami taste to a certain extent. Moreover, adding probiotics also significantly increased the content of SFA, MUFA, and PUFA in *C. nasus* muscle, particularly enhancing EPA and DHA levels. EPA and DHA are essential unsaturated fatty acids crucial for human health and development [29]. Taken together, the above results indicated that adding probiotics promoted growth performance and improved muscle nutritional value in *C. nasus*.

As an essential organ regulating lipid metabolism in animals, the liver is playing a crucial role in maintaining the homeostasis of lipid metabolism. SREBP1 plays a key role in lipid metabolism, including the synthesis of cholesterol, fatty acids, and phospholipids [30]. ACC regulates the rate-limiting step in fatty acid biosynthesis [31]. DGAT is essential for the absorption and storage of fat. ELOVL6 is a type of enzyme involved in fatty acid elongation and participating in the fatty acid synthesis [32]. In this study, adding probiotics downregulated the expression of *srebp1*, *acc*, *dgat*, and *elovl6* in *C. nasus* liver, which indicated that the adding probiotics inhibited fatty acid synthesis. HSL can hydrolyze triglycerides and plays an irreplaceable role in lipolysis [33]. PPARα is involved in fatty acid oxidation [34]. CPT1 functions to transfer long-chain fatty acids from the cytosol to the mitochondria for β-oxidation [35]. ACADM is involved in the β-oxidation of fatty acids. In this study, adding probiotics upregulated the expression of *hsl*, *pparα*, *cpt1*, and *acadm* in *C. nasus* liver. These results indicated that adding probiotics enhanced lipolysis and fat oxidation. Serum TGs are an important marker for assessing lipid metabolism [36]. In this study, adding probiotics reduced the serum TG concentration, aligning with the gene expression pattern associated with fat synthesis but contrasting with the gene expression pattern linked to lipolysis and fat oxidation. The liver also plays a pivotal role in glucose metabolism in animals to maintain glucose homeostasis. GCK plays an important role in the cellular uptake and utilization of glucose [37]. PFK is involved in the glycolytic pathway as a key enzyme [38]. In this study, adding probiotics upregulated the expression of *gck* and *pfk* in *C. nasus* liver, which indicated that adding probiotics enhanced glycolysis. G6PC is a key enzyme in maintaining glucose homeostasis and playing a role in gluconeogenesis. PEPCK is the rate-limiting enzyme of hepatic gluconeogenesis [39]. In this study, adding probiotics upregulated the expression of *g6pca1*, *g6pca2*, and *pck* in *C. nasus* liver, which suggested that adding probiotics inhibited gluconeogenesis. Serum glucose is an important indicator of glucose metabolism. In this study, adding probiotics decreased serum glucose concentration. These results indicated that the inhibited gluconeogenesis and enhanced glycolysis led to decreasing serum glucose. Furthermore, in this study, adding probiotics upregulated the expression of *ampkα* in *C. nasus* liver. AMPK is a key molecule regulating biological energy metabolism and serves as the master switch of energy metabolism [40,41]. Activation of AMPK can increase cellular uptake and utilization of glucose, promote the oxidative metabolism of glucose, suppress hepatic gluconeogenesis, and lower serum glucose levels [42]. In addition, activation of AMPK can promote the oxidative metabolism of fatty acids, reducing intracellular fat accumulation. On the other hand, activation of AMPK can also inhibit the process of fatty acid synthesis, reducing fat synthesis and storage [43]. Based on the above results, we speculated that adding probiotics activated ampkα to promote lipolysis and fat oxidation, as well as glycolysis, while inhibiting fat synthesis and gluconeogenesis.

Probiotic administration can improve the intestinal microbial community structure and function and intestinal microbial balance. In this study, adding probiotics significantly increased the relative abundance of Firmicutes in the gut. Similar results were observed in *Procambarus clarkii* feeding *Lactobacillus*, while opposite results were observed in *P. clarkii* feeding *Bacillus* [44]. Functional analysis revealed that adding probiotics significantly upregulated the relative abundance of CAZymes. The gut microbiota harbors a diverse and extensive CAZy repertoire, which facilitates the formation or degradation of oligosaccharides and polysaccharides [45]. CAZymes can also regulate the glycosylation of proteins, lipids, and complex carbohydrates [46]. Furthermore, KEGG enrichment analysis revealed that adding probiotics increased the relative abundance of genes associated with metabolism, such as carbohydrate, lipid, nucleotide, and amino acid metabolism. Previous studies have reported that adding *L. plantarum* enhanced the relative abundance of proteins associated with carbohydrate metabolism in *Siniperca chuatsi* [47]. Moreover, it has been shown that probiotics can produce some amino acids through promoting their metabolism, which can be absorbed by the host’s gut as nutrients [44,47]. The gut microbiota engages in intricate and dynamic metabolic processes within the intestine, supplying energy and nutrients for its own growth and reproduction and generating numerous metabolites for the host. These metabolites have positive effects on the host’s health, including SCFAs, bile acids, etc. [48]. In this study, metabolomic analysis revealed that adding probiotics increased the concentrations of SCFAs and bile acids. These results suggest that adding probiotics can improve the gut microbiota community structure and promote microbial metabolism. Additionally, in this study, correlation analysis was performed to investigate the relationship between gut microbiota and metabolites. The results indicated a significant positive correlation between propanoic acid and taurocholic acid with the majority of microbes at the genus level. In humans, SCFAs were produced by *Faecalibacterium*, *Lachnospira*, *Ruminococcus*, *Clostridium*, *Lactobacillus*, and others [49]. This is different from our results, which may be caused by different fermentation substrates and microbiota composition. SCFAs are generated by the gut microbiota via the fermentation of carbohydrates, proteins, and amino acids [50]. Furthermore, in this study, metabolites were significantly positively correlated with genes related to glucolipid metabolism. It has been proven that the gut and the liver are interconnected, and the gut microbiota plays a role in liver lipid and glucose metabolism through the microbiome–gut–liver axis [51,52,53]. Propionic acid is produced by the gut microbiota through the fermentation of dietary fiber. It can regulate lipid and glucose metabolism via the gut–liver axis. In the liver, propionic acid primarily undergoes gluconeogenesis and inhibits cholesterol synthesis and fat synthesis [54]. Adding 0.5% and 1% SCFAs to the feed of juvenile common carp effectively regulated glucose metabolism pathways, including glycolysis, gluconeogenesis, glycogen synthesis, and glycogenolysis after intestinal absorption and transport to the liver [55]. Sodium propionate can reduce gluconeogenesis by attenuating the gene expression of gluconeogenesis, including G6Pase and PEPCK, through activatid AMPK [56]. The gut microbiota regulates the bile acid metabolism of fish, thereby participating in lipid metabolism [57]. Dietary bile acids reduce the relative expression of lipid catabolism genes in the liver of grass carp (*Ctenopharyngodon idella*) [58]. Dietary bile acids decreased the transcription of lipid synthesis genes but increased the transcription of genes related to lipid digestion in tiger puffers (*Takifugu rubripes*) [59]. Injection of bile acids can inhibit hepatic gluconeogenesis by suppressing PEPCK and G6Pase [60,61]. Based on the above results, we speculated that adding probiotics improved gut microbiota and metabolism to participate in hepatic glucolipid metabolism through the microbiota–gut–liver axis in *C. nasus*.

Probiotics have the potential to enhance growth performance and improve nutritional value through the regulation of glucose and lipid metabolism via the gut–liver axis. Adding probiotics can promote beneficial microorganisms and metabolites (such as SCFAs) in the gut, thereby regulating glucose and lipid metabolism in the liver, subsequently impacting energy utilization and nutrient absorption in animals [62]. In this study, the mantel test analysis indicated a significant correlation between growth performance as well as nutritional value (HAAs and FFAs) and microbiota–gut–liver axis (gut microbiota, metabolites, and glucolipid metabolism). The *Pediococcus pentosaceus PP04* can significantly attenuate the increase in body weight and body fat induced by a high-fat diet in mice, downregulate levels of TG and LDL-C, and inhibit lipid accumulation in the liver [63]. Probiotics can activate hepatic AMPK signaling to promote lipolysis and inhibit fat synthesis, thereby improving the imbalance in gut microbiota induced by a high-fat diet. They also enhance the relative abundance of beneficial bacteria while suppressing the relative abundance of harmful bacteria, leading to a significant increase in concentrations of SCFAs [64]. These results supported the possibility that adding probiotics enhanced growth performance and improved nutritional value through regulating glucolipid metabolism via the gut–liver axis in *C. nasus*.

## 4. Materials and Methods

### 4.1. Experimental Animals and Sample Collection

Healthy *C. nasus* (10.14 ± 1.23 cm, 5.18 ± 0.74 g) were obtained from Yangzhong, China. Approximately 2700 *C. nasus* were randomly and equally distributed into nine pond (160 m^3^), with a final density of 300 fish per ponds. The fish were acclimatized for 7 days before the experimental study. The nine ponds were divided into three groups as follows: control group (C), probiotic supplementation in diet group (PF), and probiotic supplementation in water group (PW). In the C group, no probiotics were provided in the water and basal diet; in the PF group, 1.0 × 10^8^ CFU/g compound probiotics (*Lactobacillus plantarum*: *Saccharomyces cerevisiae*, 9:1) was provided in the basal diet (per day) [21,65,66]. The daily feed ration is 2% of the fish body weight; in the PW group, 1.0 × 10^8^ CFU/L compound probiotics was provided in the aquatic water (per week). The *L. plantarum* (1.16089) and *S. cerevisiae* (2.3973) used in this study were provided by the China General Microbiological Culture Collection Center (CGMCC). The *L. plantarum* were cultured in LB media at 28 °C for 24 h. The *S. cerevisiae* were cultured in YPD media at 28 °C for 48 h. Drum mixers were used to spray the necessary quantity of the bacterial suspension onto the diet. The experimental duration spanned 120 days (from April to August), during which continuous microbubble aeration was utilized to guarantee dissolved oxygen. Sampling was carried out at intervals of 30 days. After anesthetizing with 50 mg/L MS-222, 30 fish from each group were randomly collected. The body length and weight of the fish were recorded. Blood samples were incubated at 4 °C for 2 h, then centrifuged at 3500× *g* r/min for 10 min at 4 °C to obtain plasma. The intestinal contents were dissolved with 1% PBS and subsequently centrifuged at 4000× *g* rpm, 4 °C, for 10 min. The precipitate is immediately frozen in liquid nitrogen for the metabolome and metagenome sequencing. The liver and muscle tissues were rapidly collected and flash-frozen in liquid nitrogen, then stored at −80 °C until further analysis. The diets used in this experiment were purchased from Fentian Biotechnology Co., Ltd., Jinan, China. The detailed contents are shown in Table S1.

Various parameters, including specific growth rate, viscerosomatic index, hepatosomatic index, and condition factor, were meticulously recorded and calculated using the following equations:

$$specific\;growth\;rate,\%/d=\frac{lnFBW-lnIBW}{experiment\;period,d}\times 100$$

$$viscerosomatic\;index,\%=\frac{viscera\;weight,g}{body\;weight,g}\times 100$$

$$hepatosomatic\;index,\%=\frac{liver\;weight,g}{body\;weight,g}\times 100$$

$$condition\;factor,\%=\frac{body\;weight,g}{{\left(body\;length,cm\right)}^{3}}\times 100$$

### 4.2. Gut Microbiome Analysis Based on Metagenome Sequencing

For this, 18 fish were randomly selected from each group for intestinal microbiota analysis (three fish per sample). The DNA of six intestinal content samples of each group was extracted using the HiPure Tissue & Blood DNA Kit (Magen, Guangzhou, China). Here, 2.0 μg of DNA meeting the quality requirement was fragmented. After repairing the ends, adding a 3′ A tail, adapter ligation, and purifying the products, the DNA fragments were used to construct sequencing libraries. The libraries underwent sequencing on the Illumina NovaSeq high-throughput sequencing platform (Illumina, San Diego, CA, USA) using the PE150 strategy (Beijing, China). The clean reads were generated via filtering low-quality sequences and removing host genome sequences (GCA_007927625.1) using Fastp and bowtie2 software. The assembly was performed using QUAST software https://quast.sourceforge.net/ (accessed on 10 November 2024). After removing redundant sequences (similarity threshold 95% and coverage threshold 90%) using CD-hit software https://sites.google.com/view/cd-hit/home?authuser=0 (accessed on 10 November 2024), nonredundant gene sets were generated and annotated using the Nr, GO, KEGG, eggNOG, Pfam, SwissProt, and carbohydrate-active enzyme (CAZy) databases and the Comprehensive Antibiotic Research Database (CARD). The SILVA ribosomal RNA database was used for taxonomy annotation.

### 4.3. Metabolome of Intestinal Contents Based on LC/MS

For this, 18 fish were randomly selected from each group for intestinal metabolome analysis (three fish per group). In brief, the extract of six intestinal content samples from each group was mixed with 1000 μL of internal standard, vortexed, and mixed for 30 s. Then, they were treated using a 45 Hz grinder and ultrasonicator for 10 min (in an ice water bath). A Waters Acquity I-Class PLUS ultra-high performance liquid chromatography–mass spectrometer was used to isolate and detect metabolites using the positive ion mode (PIM) and negative ion mode (NIM). The original peak area was generated via analyzing the peak extraction, peak alignment, and other data using Progenesis QI software https://www.nonlinear.com/progenesis/qi/ (accessed on 10 November 2024), which was then normalized to the total peak area for further analysis. We used PCA and Spearman correlation to determine the repeatability of the analysis. The identified compounds were annotated using the KEGG, HMDB, and lipidmaps databases. Orthogonal projections to latent structures–discriminant analysis (OPLS-DA) modeling was conducted using the R language package ropls, and the reliability of the model was verified by performing 200 permutations. Multiple cross-validation was used to calculate the variable importance in projection (VIP) value. The differential metabolites should have a *p* value < 0.05, Foldchange > 1, and VIP > 1. Then, they were tested for enrichment of KEGG pathways using the hypergeometric distribution test.

### 4.4. Detection of Biochemical Indexes of Serum

Nine fish were randomly selected from each group for biochemical index analysis (three fish per group). The activity of CAT, SOD, ALT, and AST was detected using commercial test kits (Nanjing Jiancheng Bioengineering Institute, Nanjing, China) in accordance with the manufacturer’s instructions. The CHO, LDL-C, and MDA were detected using commercial test kits (Nanjing Jiancheng Bioengineering Institute, Nanjing, China) following the manufacturer’s instructions. The concentrations of GLU and TG were detected using commercial test kits (Beijing Solarbio Science & Technology Co., Ltd., Beijing, China) following the manufacturer’s instructions.

### 4.5. Detection of Hydrolyzed Amino Acids (HAAs) and Free Fatty Acids (FAAs)

For this, 30 fish were randomly selected from each group for HAAs and FAAs analysis (ten fish per group). The mixed standard of mother liquor (created by amino acid standard and methanol) was diluted by 10% formic acid methanol-water (1:1, *v*/*v*) to prepare the working standard solution. Weigh an appropriate amount of the isotope standard (Trp-d3), and prepare the internal standard mother solution with 10% formic acid methanol-water 1:1 to a concentration of 1000 ng/mL. Samples were added to 600 μL of 10% formic acid in methanol-water with vortex for 30 s and grinding at 55 Hz for 90 s. After centrifuging (12,000× *g* rpm, 4 °C, 5 min), 100 μL of supernatants were added to 100 μL Trp-d3 (20 ng/mL), and then the supernatant was filtered through a 0.22 μm membrane. ACQUITY UPLC BEH C18 Column (2.1 × 100 mm, 1.7 μm, Waters, Milford, MA, USA) was used for liquid chromatography. Electrospray ionization (ESI) source (positive ionization mode) was used for mass spectrum. Scans were performed using multiple reaction monitoring (MRM).

Samples were accurately weighed and added to 1 mL chloroform methanol (2:1) solution, and then they were shaken at 55 Hz for 1 min on the high-throughput tissue grinder. After centrifuging (12,000× *g* rpm, 4 °C, 5 min), all the supernatants were added to a 2 mL 1% sulfuric acid methanol solution, which was in the water bath at 80 °C for 30 min. After esterification, 1 mL n-hexane and 5 mL H_2_O (4 °C) were added for extraction and washing. After centrifuging (3500× *g* rpm, 4 °C, 5 min), 100 mg anhydrous sodium sulfate powder was added into 700 μL supernatants to remove excess water. Finally, 15 μL methyl salicylate (500 ppm) was added as an internal standard to the supernatant. The trace 1300 gas chromatograph (Thermo Fisher Scientific, Waltham, MA, USA) was used for gas chromatography analysis. ISQ 7000 (Thermo Fisher Scientific, USA) was used for mass spectrometric detection with electron impact ionization mode.

### 4.6. Real-Time Quantitative PCR Analysis

Nine fish were randomly selected from each group for RT-qPCR analysis (three fish per group). RT-qPCR was used to detect expression of genes related to glucolipid metabolism in the liver, which was performed on the Bio-Rad CFX96 real-time PCR system. Primer Premier 5 software was used to design the primers (Table S2) for RT-qPCR according to genomic coding sequences (PRJNA421870). β-actin was used for the housekeeper gene. All samples were analyzed in triplicate, and the expression of target genes was calculated using the 2^−ΔΔCT^ method.

### 4.7. Data Analysis

A two-way ANOVA was performed on SPSS 20.0. All results were presented as mean ± standard error (SE). A significance level of *p* < 0.05 was considered statistically significant. Pearson correlation analysis was employed for the correlation analysis. Statistical significance was considered at * *p* < 0.05, ** *p* < 0.01, or *** *p* < 0.001. Histograms were generated using GraphPad Prism 10.0. Statistical analysis was conducted utilizing the Biozeron Cloud Platform (http://www.cloud.biomicroclass.com/CloudPlatform (accessed on 10 November 2024)) [67]. The heatmaps were drawn via TBtools https://bio.tools/tbtools (accessed on 10 November 2024) [68].

## 5. Conclusions

The collective results displayed that adding probiotics to diet and water promoted growth performance and improved muscle nutritional value in *C. nasus*. Furthermore, adding probiotics activated *ampkα* to promote lipolysis and fat oxidation, as well as glycolysis, while inhibiting fat synthesis and gluconeogenesis. Adding probiotics can reconstruct intestinal microbiota community structure and reprogram the metabolism of intestinal microbiota. They regulated hepatic glucolipid metabolism through the microbiota–gut–liver axis. Mantel test analysis implied that adding probiotics promoted growth performance and improved muscle nutritional value via improving gut–liver axis. Our findings provide novel insights into the mechanisms underlying the enhancement of growth performance and nutritional value in *C. nasus* and other fish by adding probiotics. Our results will also contribute to a further comprehension of the interaction mechanisms between probiotics and the gut–liver axis, offering a scientific foundation for the utilization of probiotics in aquaculture.

## Figures and Tables

**Table 1:** Effects of probiotic supplementation on the growth performance of *C. nasus*.

Index	30d			60d			90d			120d		
	C	PW	PF	C	PW	PF	C	PW	PF	C	PW	PF
FBW	6.93 ± 1.57 ^bB^	8.38 ± 1.40 ^aB^	8.92 ± 2.96 ^aD^	8.31 ± 2.62 ^bB^	9.51 ± 1.90 ^abAB^	10.95 ± 2.39 ^aC^	9.18 ± 1.92 ^bB^	10.23 ± 3.59 ^abAB^	12.64 ± 3.47 ^aB^	10.49 ± 3.26 ^bA^	11.25 ± 2.74 ^bA^	16.44 ± 4.27 ^aA^
BL	12.38 ± 1.2 ^aC^	13.01 ± 0.77 ^aC^	13.21 ± 1.36 ^aC^	13.27 ± 1.30 ^bBC^	14.00 ± 1.01 ^abB^	14.77 ± 1.14 ^aB^	13.86 ± 1.18 ^bB^	14.91 ± 1.65 ^aB^	14.95 ± 1.71 ^aB^	16.20 ± 1.46 ^bA^	17.03 ± 1.29 ^abA^	17.65 ± 1.81 ^aA^
SGR	1.00 ± 0.78 ^bA^	1.68 ± 0.55 ^aA^	1.77 ± 1.01 ^aA^	0.83 ± 0.43 ^bAB^	1.04 ± 0.33 ^abAB^	1.27 ± 0.36 ^aA^	0.55 ± 0.23 ^bB^	0.74 ± 0.33 ^aB^	0.79 ± 0.35 ^aB^	0.58 ± 0.23 ^b^	0.65 ± 0.20 ^ab^	0.85 ± 0.25 ^a^
VSI	8.08 ± 2.69 ^A^	7.27 ± 2.04 ^A^	8.27 ± 2.48 ^A^	6.86 ± 2.40 ^AB^	8.15 ± 3.00 ^A^	7.73 ± 2.19 ^A^	6.90 ± 1.81 ^AB^	6.27 ± 1.70 ^AB^	6.13 ± 0.78 ^B^	5.78 ± 1.36 ^B^	5.34 ± 1.67 ^B^	6.61 ± 1.85 ^B^
HSI	0.82 ± 0.27 ^A^	0.89 ± 0.44 ^A^	1.17 ± 0.39	0.52 ± 0.14 ^B^	0.64 ± 0.36 ^AB^	0.55 ± 0.22	0.63 ± 0.19 ^AB^	0.51 ± 0.16 ^B^	0.50 ± 0.19	0.59 ± 0.25 ^AB^	0.51 ± 0.18 ^B^	0.64 ± 0.25
CF	0.36 ± 0.04 ^A^	0.38 ± 0.03 ^A^	0.38 ± 0.02 ^A^	0.35 ± 0.04 ^A^	0.34 ± 0.03 ^B^	0.34 ± 0.03 ^B^	0.30 ± 0.02 ^B^	0.30 ± 0.02 ^C^	0.31 ± 0.03 ^C^	0.24 ± 0.02 ^B^	0.22 ± 0.01 ^D^	0.26 ± 0.02 ^D^

Note: FBW, final body weight; BL, body length; SGR, specific growth rate; VSI, viscerosomatic index; HSI, hepatosomatic index; CF, condition factor. The values were shown in means ± SE. *n* = 30 per treatment group. Different capital letters indicate significant differences among different time points in the same groups at *p* < 0.05. Different lower-case letters indicate significant differences between different groups at the same time point at *p* < 0.05.

**Table 2:** Effects of probiotic supplementation on hydrolyzed amino acids.

	C30d	PF30d	PW30d	C60d	PF60d	PW60d	C90d	PF90d	PW90d	C120d	PF120d	PW120d
Gly	1197.85 ± 26.68 Ab	1329.69 ± 21.91 Aa	1265.83 ± 62.48 Aab	1075.87 ± 63.28 Aa	1084.03 ± 38.08 ABa	833.03 ± 40.11 Ba	866.61 ± 38.37 Bc	934.19 ± 24.43 Bb	1140.72 ± 41.99 Aa	1001.01 ± 28.98 Aa	892.79 ± 36.64 Bb	1096.13 ± 36.44 Aa
Ala	204.69 ± 10.41 Bb	229.96 ± 9.55 Ba	136.51 ± 16.21 Cc	236.90 ± 9.27 ABa	243.19 ± 15.31 ABa	219.96 ± 14.59 Ba	183.27 ± 5.64 Bb	209.37 ± 12.93 Bb	297.32 ± 8.05 Aa	298.75 ± 29.98 Aab	289.44 ± 1.72 Ab	311.03 ± 9.94 Aa
Ser	232.87 ± 5.02 Ab	319.79 ± 13.61 Aa	300.75 ± 4.23 Aa	134.22 ± 5.69 Ba	156.62 ± 6.64 Ba	138.42 ± 8.98 Ca	84.23 ± 0.43 Cc	114.19 ± 1.48 Cb	191.83 ± 1.46 Ba	150.27 ± 5.17 Ba	87.66 ± 3.93 Db	182.59 ± 7.62 Ba
Pro	86.85 ± 6.05 ABc	128.75 ± 9.46 Ab	204.17 ± 7.27 Aa	55.45 ± 0.78 Bb	63.91 ± 3.65 Bab	85.62 ± 11.88 Ca	62.62 ± 1.24 Bb	87.26 ± 9.96 Bb	124.99 ± 4.97 Ba	104.01 ± 8.20 Aab	98.56 ± 3.71 ABb	140.84 ± 6.77 Ba
Asn	38.86 ± 2.01 Aa	25.14 ± 1.63 Ab	22.89 ± 1.45 Ab	15.06 ± 1.53 Bb	21.62 ± 1.49 Aa	22.66 ± 1.80 Aa	29.29 ± 1.11 Aa	27.23 ± 2.96 Aa	26.44 ± 2.83 Aa	6.39 ± 0.35 Cb	13.03 ± 0.64 Ba	11.91 ± 0.84 Ba
Asp	19.87 ± 1.02 Bab	16.77 ± 0.67 Cb	23.50 ± 0.20 Aa	28.49 ± 1.42 Aa	25.41 ± 2.59 Ba	23.88 ± 1.95 Aa	32.26 ± 1.29 Ab	44.01 ± 0.96 Aa	28.76 ± 0.57 Ab	24.76 ± 1.68 ABa	24.90 ± 0.44 Ba	27.33 ± 2.01 Aa
Gln	76.32 ± 2.51 Ba	34.77 ± 2.11 Cb	31.56 ± 0.41 Db	72.71 ± 2.19 Bb	88.53 ± 3.59 Bb	106.81 ± 5.94 Ba	125.90 ± 1.65 Ab	133.87 ± 2.39 Aa	136.98 ± 2.94 Aa	79.90 ± 3.88 Bb	114.50 ± 3.59 Aa	78.24 ± 5.15 Cb
Glu	49.53 ± 2.94 Ca	37.59 ± 1.72 Cb	35.01 ± 1.56 Cb	140.38 ± 8.87 Aa	147.97 ± 9.74 Ba	129.45 ± 10.31 Ba	165.04 ± 3.98 Ac	204.44 ± 1.97 Ab	258.81 ± 3.09 Aa	91.66 ± 7.89 Bc	121.86 ± 5.47 Bb	150.99 ± 8.81 Ba
His	285.60 ± 15.87 Ab	354.51 ± 26.49 Aa	337.13 ± 23.33 Aa	155.88 ± 11.14 Bb	184.48 ± 19.54 Ca	196.75 ± 27.36 Ba	189.13 ± 26.29 Bb	221.45 ± 13.55 Ba	204.48 ± 15.01 Bab	232.94 ± 35.00 Aa	171.62 ± 8.07 Cb	216.86 ± 13.16 Ba
Arg	9.73 ± 0.51 Cb	10.07 ± 0.61 Cb	16.11 ± 0.97 Ca	18.68 ± 0.87 ABb	30.61 ± 2.83 Aa	31.33 ± 4.11 Aa	14.47 ± 1.65 Bb	26.65 ± 1.08 Aa	24.58 ± 1.22 Ba	23.38 ± 3.10 Aa	16.20 ± 0.80 Bb	16.32 ± 1.06 Cb
Tyr	16.59 ± 0.49 Bb	23.76 ± 1.41 Aa	24.75 ± 0.84 Aa	25.08 ± 1.10 Aa	23.79 ± 1.62 Aab	20.06 ± 0.12 Ab	17.25 ± 0.52 Bb	20.69 ± 0.99 Aa	20.73 ± 0.73 Aa	23.44 ± 0.44 Aa	19.41 ± 1.07 Aa	23.47 ± 0.75 Aa
Val	32.64 ± 0.14 Ba	36.21 ± 2.22 BCa	35.79 ± 2.94 Ba	42.15 ± 1.69 Ab	50.79 ± 1.41 Aa	45.46 ± 1.28 ABab	31.18 ± 0.37 Bc	41.72 ± 1.67 Bb	51.70 ± 0.74 Aa	38.35 ± 0.19 ABb	27.58 ± 1.46 Cc	54.40 ± 3.20 Aa
Thr	28.61 ± 3.06 Cb	48.43 ± 3.50 Aa	48.45 ± 1.10 Aa	36.67 ± 1.29 Bb	45.08 ± 4.79 Aa	48.48 ± 5.16 Aa	26.02 ± 1.42 Cc	38.59 ± 2.22 Bb	53.64 ± 1.42 Aa	45.87 ± 6.18 Aa	37.93 ± 1.03 Bb	49.66 ± 3.79 Aa
Ile	20.35 ± 0.37 Ba	24.34 ± 1.02 Ba	23.24 ± 1.38 Ba	27.58 ± 1.24 Aa	31.85 ± 1.09 Aa	26.94 ± 0.70 Ba	18.41 ± 0.52 Bc	24.29 ± 0.63 Bb	30.36 ± 0.38 ABa	23.21 ± 0.27 ABb	16.25 ± 1.06 Cc	32.10 ± 1.72 Aa
Leu	31.15 ± 0.06 Aa	35.66 ± 2.14 Ba	34.94 ± 1.61 Ba	38.83 ± 1.54 Ab	45.96 ± 2.60 Aa	36.30 ± 0.73 Bb	26.51 ± 0.47 Bc	36.90 ± 1.11 Bb	43.33 ± 0.73 ABa	33.10 ± 0.42 Ab	28.66 ± 1.81 Cb	47.65 ± 1.98 Aa
Lys	3.32 ± 0.07 Ba	3.91 ± 0.22 BCa	3.89 ± 0.28 Ba	2.97 ± 0.14 Ba	2.95 ± 0.17 Ca	2.47 ± 0.16 Ca	3.52 ± 3.24 Bb	5.52 ± 0.19 Ba	2.69 ± 0.05 Cb	5.11 ± 0.47 Ab	11.79 ± 0.65 Aa	5.14 ± 0.55 Ab
Met	21.70 ± 0.13 Bb	28.88 ± 1.01 Aa	27.13 ± 1.70 ABa	29.97 ± 0.55 Aa	27.52 ± 0.36 Aa	24.01 ± 0.97 Ba	24.65 ± 0.27 Ba	27.75 ± 1.26 Aa	29.27 ± 0.15 Aa	31.71 ± 0.46 Aa	26.45 ± 0.99 Ab	33.58 ± 1.06 Aa
Phe	19.88 ± 0.46 Bb	25.21 ± 0.75 Aa	26.19 ± 1.32 Aa	28.21 ± 0.98 Aa	26.28 ± 1.13 Aa	19.37 ± 0.61 Bb	20.04 ± 0.68 Ba	24.47 ± 0.73 Aa	22.61 ± 0.10 ABa	24.42 ± 0.27 ABa	19.85 ± 1.14 Aa	25.27 ± 0.73 ABa
Trp	7.14 ± 0.12 Bb	8.73 ± 0.51 ABab	9.43 ± 0.49 Aa	9.71 ± 1.03 Aa	8.65 ± 0.38 ABab	7.05 ± 0.30 Bb	7.88 ± 0.27 Bb	9.81 ± 0.24 Aa	7.55 ± 0.27 Bb	9.31 ± 0.73 Aa	7.64 ± 0.34 Bb	7.33 ± 0.25 Bb
EAA	164.82 ± 3.35 Bb	211.41 ± 10.72 Aa	209.08 ± 8.85 Ba	216.12 ± 8.32 Aa	239.11 ± 11.64 Aa	210.11 ± 8.36 Ba	158.24 ± 1.23 Bc	209.07 ± 3.12 Ab	241.18 ± 3.06 Aa	211.11 ± 5.70 Ab	176.18 ± 6.19 Bc	255.16 ± 11.39 Aa
NEAA	2218.79 ± 40.84 Aa	2510.83 ± 72.01 Aa	2398.26 ± 30.58 Aa	1958.76 ± 95.86 ABa	2070.21 ± 100.80 Ba	1808.03 ± 119.83 Ba	1770.11 ± 73.16 Bc	2023.40 ± 14.78 Bb	2455.67 ± 46.07 Aa	2036.56 ± 120.75 ABab	1850.03 ± 58.48 Cb	2255.76 ± 87.58 ABa
TAA	2383.62 ± 43.94 Ab	2722.24 ± 82.65 Aa	2607.35 ± 34.41 Aa	2174.89 ± 102.61 ABb	2309.32 ± 112.31 Ba	2018.15 ± 128.19 Bb	1928.35 ± 74.29 Bc	2232.47 ± 15.30 Bb	2696.86 ± 44.37 Aa	2247.68 ± 126.28 ABab	2026.21 ± 64.56 Cb	2510.92 ± 98.95 Aa

Note: EAA: essential amino acids, NEAA: nonessential amino acids, TAA: total amino acid. The values were shown in means ± SE. *n* = 30 per treatment group. Different capital letters indicate significant differences among different time points in the same groups at *p* < 0.05. Different lower-case letters indicate significant differences between different groups at the same time point at *p* < 0.05.

**Table 3:** Effects of probiotic supplementation on free fatty acids.

	C30d	PF30d	PW30d	C60d	PF60d	PW60d	C90d	PF90d	PW90d	C120d	PF120d	PW120d
C6:0	0.99 ± 0.11	0.86 ± 0.04	0.95 ± 0.02	0.90 ± 0.01	0.89 ± 0.08	0.91 ± 0.02	0.93 ± 0.05	1.01 ± 0.12	0.88 ± 0.02	0.89 ± 0.02	0.92 ± 0.05	0.79 ± 0.04
C8:0	0.17 ± 0.01	0.14 ± 0.01	0.14 ± 0.01	0.13 ± 0.01	0.14 ± 0.01	0.15 ± 0.01	0.11 ± 0.01	0.13 ± 0.01	0.12 ± 0.01	0.12 ± 0.01	0.12 ± 0.01	0.13 ± 0.01
C10:0	1.13 ± 0.02 Aa	1.12 ± 0.04 Aa	1.55 ± 0.02 Aa	0.80 ± 0.02 ABb	1.03 ± 0.01 Aab	1.33 ± 0.01 ABa	0.60 ± 0.01 Bb	1.02 ± 0.12 Aa	0.81 ± 0.01 Bab	0.59 ± 0.01 Bb	0.45 ± 0.01 Bb	0.93 ± 0.01 Ba
C11:0	0.35 ± 0.01	0.34 ± 0.01	0.45 ± 0.01	0.27 ± 0.01	0.33 ± 0.01	0.39 ± 0.01	0.23 ± 0.01	0.34 ± 0.04	0.27 ± 0.01	0.25 ± 0.01	0.16 ± 0.01	0.34 ± 0.01
C12:0	4.89 ± 0.09 ABa	4.70 ± 0.12 Aa	5.96 ± 0.08 Aa	4.37 ± 0.03 Bb	5.24 ± 0.17 Aa	5.90 ± 0.08 Aa	4.20 ± 0.10 Bb	5.57 ± 0.64 Aa	4.38 ± 0.20 Bb	5.28 ± 0.11 Aa	3.31 ± 0.11 Bb	5.86 ± 0.05 Aa
C13:0	1.42 ± 0.06 Aa	1.32 ± 0.02 ABa	1.69 ± 0.04 Aa	1.20 ± 0.01 Aa	1.50 ± 0.04 Aab	1.95 ± 0.06 Aa	1.19 ± 0.02 Ab	1.68 ± 0.19 Aa	1.51 ± 0.05 Aa	1.57 ± 0.04 Aa	0.87 ± 0.02 Bb	1.84 ± 0.02 Aa
C14:0	144.26 ± 2.80 ABa	139.93 ± 2.70 Ba	172.75 ± 0.73 ABa	129.37 ± 1.80 Bb	157.11 ± 4.19 ABab	187.09 ± 2.98 ABa	113.59 ± 4.15 Bb	177.21 ± 19.03 Aa	161.90 ± 4.30 Ba	161.69 ± 3.15 Ab	99.99 ± 2.31 Cc	210.62 ± 2.31 Aa
C14:1	3.74 ± 0.18	3.43 ± 0.13	4.14 ± 0.04	3.00 ± 0.09	3.50 ± 0.09	4.11 ± 0.03	2.60 ± 0.07	3.66 ± 0.21	3.42 ± 0.05	4.29 ± 0.11	2.43 ± 0.10	4.20 ± 0.12
C15:0	14.93 ± 0.45 Aa	14.16 ± 0.18 Ba	16.83 ± 0.16 Ba	13.65 ± 0.17 Ab	16.59 ± 0.83 ABb	21.04 ± 0.26 Aa	14.03 ± 0.03 Ab	20.50 ± 2.49 Aa	18.93 ± 0.62 ABab	18.91 ± 0.38 Aa	10.43 ± 0.47 Cb	22.97 ± 0.31 Aa
C15:1	1.04 ± 0.09	1.09 ± 0.02	0.99 ± 0.02	0.98 ± 0.01	0.89 ± 0.02	1.66 ± 0.04	0.94 ± 0.02	1.08 ± 0.02	1.19 ± 0.02	1.90 ± 0.04	0.85 ± 0.05	0.75 ± 0.02
C16:0	1102.06 ± 19.01 Aa	975.48 ± 37.35 Ba	1093.18 ± 20.81 Ba	950.55 ± 11.26 ABa	1065.51 ± 40.43 ABa	1181.35 ± 41.00 Ba	870.31 ± 6.16 Bc	1201.21 ± 121.28 Aa	1097.27 ± 35.29 Bb	1065.77 ± 18.06 Ab	813.77 ± 18.37 Cc	1275.54 ± 15.75 Aa
C16:1	336.65 ± 9.02 Aab	313.66 ± 10.95 Ab	384.05 ± 11.10 Aa	262.40 ± 4.09 Ba	329.43 ± 13.29 Ab	360.95 ± 8.58 ABa	191.04 ± 3.16 Cb	326.33 ± 37.05 Aa	305.99 ± 9.52 Ba	289.99 ± 2.26 Bb	175.86 ± 5.18 Bc	393.80 ± 15.04 Aa
C17:0	14.72 ± 0.39	13.57 ± 0.31	14.80 ± 0.27	13.34 ± 0.12	16.70 ± 0.61	23.73 ± 0.61	16.12 ± 0.39	22.25 ± 3.07	21.20 ± 0.67	23.75 ± 0.20	12.04 ± 0.45	26.09 ± 0.73
C17:1	11.54 ± 2.62	11.94 ± 1.16	12.01 ± 0.89	10.86 ± 1.61	11.91 ± 0.31	15.19 ± 3.08	12.01 ± 1.19	20.26 ± 2.81	16.74 ± 1.11	15.71 ± 3.36	9.00 ± 0.96	22.46 ± 0.35
C18:0	186.39 ± 7.07 ABa	161.23 ± 5.17 Ca	189.56 ± 4.20 a	165.42 ± 1.70 Bc	197.07 ± 9.93 Bb	244.40 ± 1.94 ABa	191.02 ± 6.79 ABb	250.62 ± 24.39 Aa	222.97 ± 5.91 Bab	239.13 ± 8.23 Aa	168.50 ± 3.57 Cb	297.13 ± 3.63 Aa
C18:1	2181.16 ± 159.84 Aab	1838.44 ± 36.84 ABb	2261.85 ± 111.02 Aa	1926.65 ± 103.62 ABa	2013.97 ± 67.10 AA	2103.01 ± 154.95 ABA	1607.82 ± 116.86 Bb	1960.50 ± 161.01 ABa	1845.72 ± 56.53 Bab	1913.37 ± 112.22 ABa	1602.24 ± 38.26 Bb	1993.18 ± 59.04 Ba
C18:2	726.47 ± 15.68 Ba	607.26 ± 14.41 Cb	767.45 ± 21.22 Ca	650.00 ± 11.42 Cc	764.86 ± 48.92 Bb	948.44 ± 14.70 Ba	704.85 ± 7.95 BCc	998.51 ± 106.08 Aa	845.13 ± 8.42 Cb	918.66 ± 20.24 Ab	708.47 ± 18.18 Cc	1114.57 ± 12.07 Aa
C18:3	92.92 ± 3.45 Ba	97.97 ± 3.81 Ba	107.63 ± 1.79 Ba	89.97 ± 1.75 Bc	132.26 ± 5.57 Ab	176.75 ± 1.08 Aa	104.29 ± 1.11 ABb	148.17 ± 21.32 Aa	134.17 ± 2.98 ABa	145.52 ± 4.62 Aa	98.62 ± 3.83 Bb	172.95 ± 3.38 Aa
C19:1	5.61 ± 0.36 ABb	5.07 ± 0.12 Cb	6.88 ± 0.13 Ba	4.83 ± 0.13 Bb	6.50 ± 0.06 ABa	7.98 ± 0.19 Aa	5.55 ± 0.08 ABb	7.84 ± 0.53 Aa	6.50 ± 0.04 Bab	7.21 ± 0.02 Ab	6.08 ± 0.08 Bc	9.15 ± 0.32 Aa
C20:0	12.07 ± 0.32 Aab	10.37 ± 0.28 Bb	13.89 ± 0.70 ABa	10.01 ± 0.17 Ab	10.98 ± 0.46 Bb	15.36 ± 0.62 Aa	9.45 ± 0.26 Ab	14.91 ± 2.06 Aa	12.65 ± 0.31 Bab	12.32 ± 0.06 Ab	7.96 ± 0.31 Cc	16.73 ± 0.30 Aa
C20:1	65.28 ± 1.65 Aa	56.94 ± 1.83 BCb	69.48 ± 1.05 Ca	54.98 ± 0.80 Bc	66.10 ± 1.97 Bb	85.60 ± 0.31 Ba	54.76 ± 2.15 Bb	85.18 ± 13.32 Aa	73.03 ± 1.80 Ca	68.29 ± 2.31 Ab	44.52 ± 1.53 Cc	95.15 ± 1.35 Aa
C20:2	18.94 ± 0.86 A	16.21 ± 0.32 B	17.45 ± 0.52 B	13.87 ± 0.11 B	16.39 ± 0.83 B	18.52 ± 0.29 AB	13.26 ± 0.13 B	19.88 ± 2.37 A	16.33 ± 0.53 B	16.61 ± 0.19 A	11.61 ± 0.55 C	21.13 ± 0.20 A
C20:3	16.15 ± 1.44 ABa	13.86 ± 0.23 Ba	15.35 ± 0.39 Ba	12.92 ± 0.33 Bb	16.69 ± 0.68 ABa	19.53 ± 0.31 Aa	14.09 ± 0.35 Bb	20.00 ± 2.19 Aa	16.84 ± 0.68 Bb	18.66 ± 0.36 Aa	13.53 ± 0.58 Bb	20.79 ± 0.21 Aa
C20:4	53.94 ± 1.61 Ba	52.50 ± 1.51 Aa	44.03 ± 2.25 Cb	40.63 ± 0.66 Cc	50.16 ± 1.88 Ab	57.15 ± 2.36 Ba	50.55 ± 1.00 Ba	52.49 ± 6.06 Aa	52.16 ± 1.68 Ba	65.26 ± 3.33 Aa	37.71 ± 1.86 Bb	78.54 ± 1.92 Aa
C20:5	277.42 ± 4.56 Aa	246.40 ± 3.78 Aa	253.01 ± 11.13 Aa	173.09 ± 3.46 Bb	224.32 ± 12.41 Aa	253.79 ± 3.73 Aa	144.79 ± 3.29 Bb	207.50 ± 21.68 Aa	196.28 ± 7.95 Ba	155.55 ± 5.12 Bb	99.92 ± 6.68 Bc	222.33 ± 10.58 ABa
C21:0	1.18 ± 0.10	1.06 ± 0.02	1.36 ± 0.06	1.07 ± 0.04	1.16 ± 0.04	1.64 ± 0.01	1.09 ± 0.03	1.53 ± 0.15	1.26 ± 0.01	1.41 ± 0.01	0.94 ± 0.05	1.72 ± 0.01
C22:0	3.94 ± 0.05 Bb	3.54 ± 0.04 ABb	5.11 ± 0.17 Ba	3.65 ± 0.03 Bb	3.88 ± 0.07 Bb	6.19 ± 0.12 Aa	3.51 ± 0.07 Ba	4.81 ± 0.39 Aa	4.36 ± 0.01 Ba	5.17 ± 0.07 Aa	3.06 ± 0.10 Bb	6.88 ± 0.16 Aa
C22:1	8.62 ± 0.73 Aa	7.04 ± 0.08 Ab	8.68 ± 0.17 Aa	6.56 ± 0.14 Bb	6.73 ± 0.34 Ab	8.74 ± 0.15 Aa	6.02 ± 0.08 Bb	7.84 ± 0.55 Aa	7.73 ± 0.03 Aa	6.61 ± 0.13 Bb	4.80 ± 0.19 Bc	8.44 ± 0.08 Aa
C22:2	1.70 ± 0.30	1.27 ± 0.02	1.41 ± 0.08	1.23 ± 0.01	1.28 ± 0.06	1.52 ± 0.06	1.37 ± 0.02	1.69 ± 0.17	1.40 ± 0.03	1.40 ± 0.04	1.22 ± 0.03	1.44 ± 0.04
C22:4	5.23 ± 0.60 ABa	4.43 ± 0.14 Ba	4.57 ± 0.20 Ba	3.76 ± 0.03 Bb	4.50 ± 0.22 Ba	5.00 ± 0.08 Ba	4.86 ± 0.07 Ba	5.74 ± 0.78 Aa	4.95 ± 0.03 Ba	6.56 ± 0.13 Aab	4.18 ± 0.19 Ba	7.85 ± 0.13 Aa
C22:5	103.11 ± 4.54 Aa	89.21 ± 0.94 Ab	84.87 ± 1.83 Bb	65.88 ± 0.50 Bb	78.44 ± 2.99 Bb	91.64 ± 1.93 ABa	63.51 ± 0.95 Bb	92.99 ± 11.63 Aa	80.92 ± 0.67 Ba	71.76 ± 2.01 Bb	51.52 ± 2.37 Cc	101.43 ± 2.37 Aa
C22:6	719.14 ± 12.33 Aa	576.17 ± 20.14 Ab	503.88 ± 17.14 Ab	387.55 ± 12.70 Bb	365.44 ± 9.55 Bb	449.88 ± 15.43 ABa	330.89 ± 2.48 Bb	468.11 ± 57.17 Aa	409.77 ± 8.21 Ba	301.14 ± 8.53 Bb	217.91 ± 9.30 Cb	448.93 ± 13.90 ABa
C23:0	2.20 ± 0.07 Aa	1.66 ± 0.06 Aa	1.91 ± 0.03 Aa	1.44 ± 0.05 ABa	1.20 ± 0.01 Aa	1.64 ± 0.09 Aa	1.21 ± 0.01 Ba	1.55 ± 0.10 Aa	1.32 ± 0.03 Aa	1.76 ± 0.04 ABa	1.16 ± 0.06 Ab	1.71 ± 0.03 Aa
C24:0	3.27 ± 0.37 A	2.49 ± 0.09 B	3.29 ± 0.19 A	2.82 ± 0.04 A	2.94 ± 0.20 B	3.83 ± 0.08 A	3.28 ± 0.03 A	4.04 ± 0.38 A	3.26 ± 0.07 A	3.72 ± 0.11 A	2.17 ± 0.09 B	5.12 ± 0.14 A
C24:1	12.59 ± 1.56 Aa	9.09 ± 0.29 ABa	10.50 ± 0.37 Aa	8.52 ± 0.16 Ba	8.27 ± 0.28 ABa	10.06 ± 0.05 Aa	7.59 ± 0.26 Bb	11.81 ± 1.43 Aa	10.14 ± 0.23 Aa	9.20 ± 0.26 ABab	6.44 ± 0.28 Bb	12.20 ± 0.24 Aa
ΣSFA	1494.04 ± 28.01 Aa	1332.04 ± 45.96 Bb	1523.49 ± 27.12 Ba	1299.07 ± 14.57 Bb	1482.37 ± 56.91 ABab	1696.96 ± 46.80 ABa	1230.96 ± 15.00 Bb	1708.45 ± 173.35 Aa	1553.16 ± 45.87 Ba	1542.40 ± 23.08 Aa	1125.91 ± 25.68 Cb	1874.47 ± 22.48 Aa
ΣMUFA	2626.28 ± 170.89 Aa	2246.75 ± 48.62 Aa	2758.62 ± 122.08 Aa	2278.82 ± 99.14 ABa	2447.33 ± 82.87 Aa	2597.33 ± 160.46 Ba	1888.36 ± 118.81 Bb	2424.54 ± 215.15 Aa	2270.49 ± 66.82 Cab	2316.60 ± 107.97 Aa	1852.27 ± 43.16 Bb	2539.36 ± 72.65 Ba
ΣPUFA	2015.08 ± 44.74 Aa	1705.33 ± 44.92 Bb	1799.70 ± 55.42 Bb	1438.92 ± 4.79 Bb	1654.39 ± 79.22 Bb	2022.25 ± 32.67 ABa	1432.50 ± 16.45 Bb	2015.11 ± 229.34 Aa	1757.98 ± 26.64 Bab	1701.17 ± 39.53 ABb	1244.75 ± 42.51 Cc	2190.00 ± 34.67 Aa

Note: SFA: saturated fatty acids, MUFA: monounsaturated fatty acids, PUMA: polyunsaturated fatty acids. The values were showed in means ± SE. *n* = 30 per treatment group. Different capital letters indicate significant differences among different time points in the same groups at *p* < 0.05. Different lower-case letters indicate significant differences between different groups at the same time point at *p* < 0.05.

## Data Availability

The sequences were submitted to the NCBI SRA database (PRJNA 1102166, https://www.ncbi.nlm.nih.gov/bioproject/PRJNA1102166 (accessed on 10 November 2024)).

## Supplementary Materials

The supporting information can be downloaded at: https://www.mdpi.com/article/10.3390/ijms252212196/s1.

## Abbreviations

FBW: final body weight, BL: body length, SGR: specific growth rate, EAAs: essential amino acids, TAAs: total amino acids, SFAs: saturated fatty acids, MUFAs: monounsaturated fatty acids, PUFAs: polyunsaturated fatty acids, SCFAs: short-chain fatty acids, VSI: viscerosomatic index, HSI: hepatosomatic index, CF: condition factor, NEAA: nonessential amino acids, EAA: essential amino acids, TAA: total amino acid, SFA: saturated fatty acids, MUFA: monounsaturated fatty acids, PUMA: polyunsaturated fatty acids, CAZyme: carbohydrate-active enzymes, LEfSe: linear discriminant analysis effect size, LDA: linear discriminant analysis, SCFAs: short-chain fatty acids, srebp1: sterol-regulatory element-binding protein 1, acc: acetyl-coa carboxylase, dgat: diacylglycerol acyltransferase, elvol6: elongation of very long-chain fatty acids protein 6, hsl: hormone-sensitive lipase, pparα: peroxisome proliferators-activated receptors, cpt1: carnitine palmitoyltransferase 1, acadm: acyl-coa dehydrogenase, gck: glucokinase, pfk: phosphofructokinase, g6pca1: glucose-6-phosphatase catalytic subunit, pck: phosphoenolpyruvate carboxykinase, ampkα: adenosine 5′-monophosphate (AMP)-activated protein kinase α, G6Pase: glucose-6-phosphatase, PEPCK: phosphoenolpyruvate carboxykinase, CAT: catalase, SOD: superoxide dismutase, ALT: alanine aminotransferase, AST: aspartate aminotransferase, CHO: cholesterol, LDL-C: low-density lipoprotein cholesterol, MDA: malondialdehyde, GLU: glucose, TG: triglyceride.
