# Supplementary material for: Probiotics Enhance Coilia nasus Growth Performance and Nutritional Value by Regulating Glucolipid Metabolism via the Gut–Liver Axis
Source: Int J Mol Sci. 2024 Nov 13;25(22):12196. doi: 10.3390/ijms252212196 (PMC11594500; doi:10.3390/ijms252212196)
Supplement: Supplementary file 1 [file ijms-25-12196-s001.zip › supplementary materials/supplementary materials figure S1-S5.pdf]

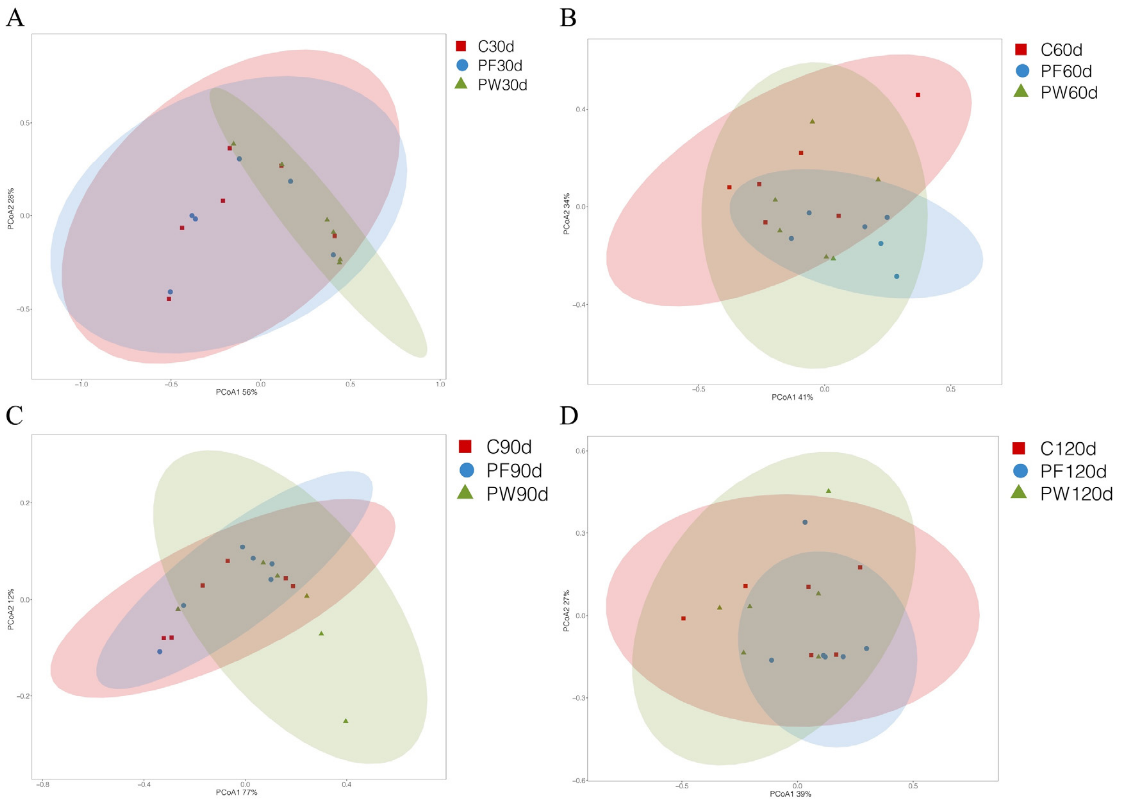

Figure S1. PCoA analysis of *C. nasus* intestinal microbiota affected by probiotics supplementation at 30d (A), 60d (B), 90d (C), and 120d (D).

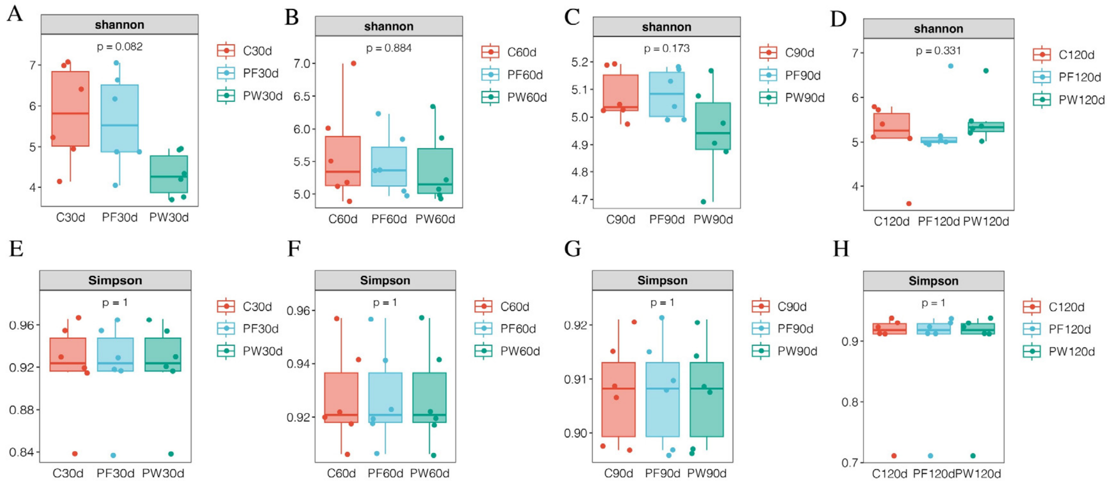

Figure S2. α diversity analysis. Shannon indexes of *C. nasus* intestinal microbiota affected by probiotics supplementation at 30d (A), 60d (B), 90d (C), and 120d (D). Simpson indexes of *C. nasus* intestinal microbiota affected by probiotics supplementation at 30d (E), 60d (F), 90d (G), and 120d (H).

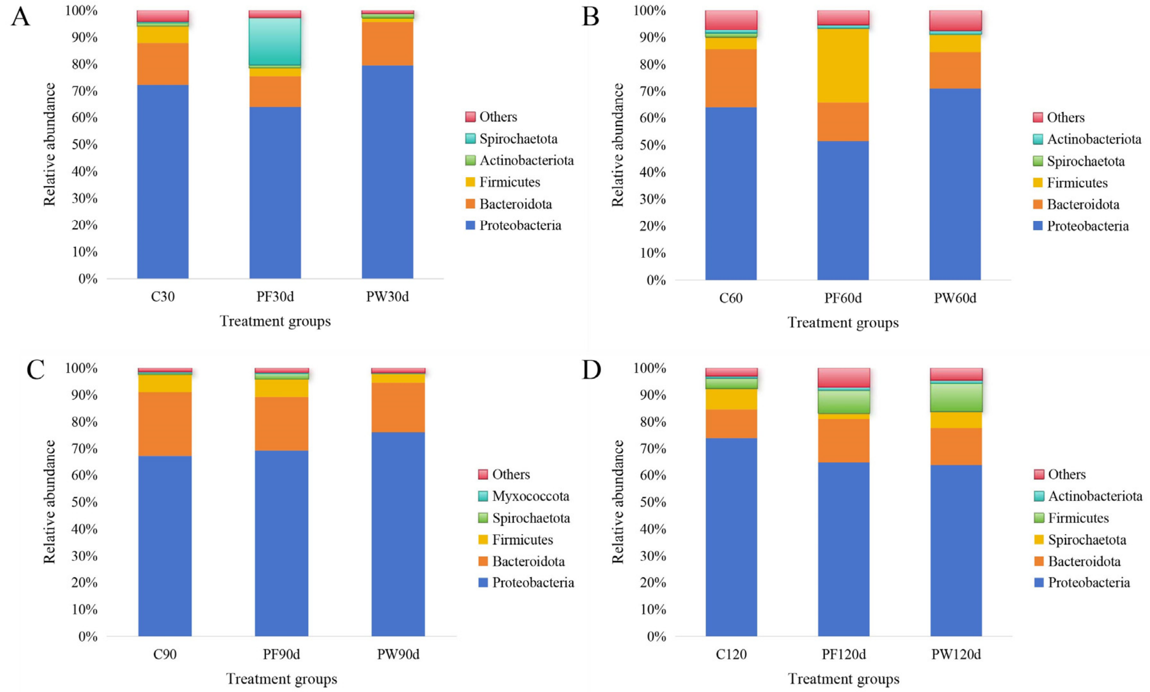

Figure S3. Gut microbiota community structure analysis of *C. nasus* intestinal microbiota affected by probiotics supplementation at 30d (A), 60d (B), 90d (C), and 120d (D).

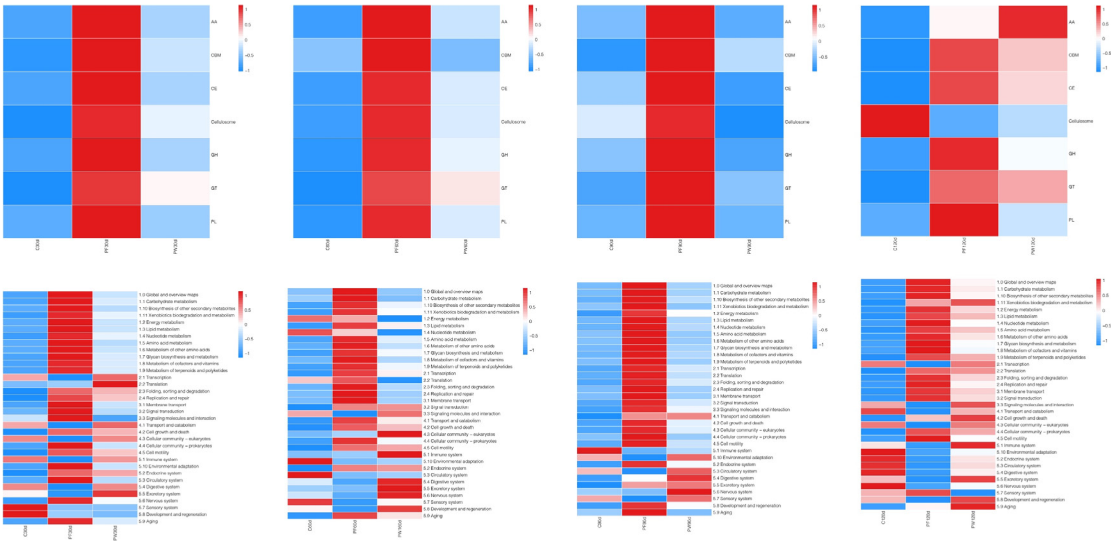

Figure S4. CAZy and KEGG analysis of *C. nasus* intestinal microbiota affected by probiotics supplementation.

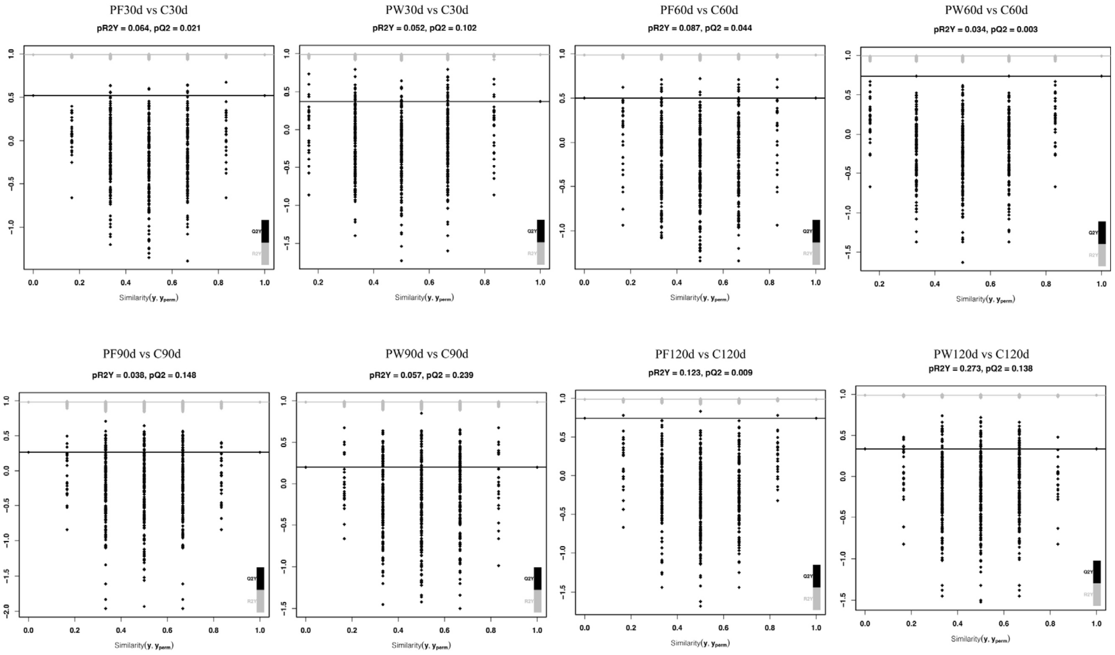

Figure S5. OPLS-DA analysis of *C. nasus* intestinal microbiota metabolites affected by probiotics supplementation.
